# Seasonal variations in nocturnal changes in blood pressure between Ireland and Singapore

**DOI:** 10.1016/j.ctrsc.2015.10.006

**Published:** 2015-11-02

**Authors:** Lin Ho Wong, Peter Ting, David Kerins

**Affiliations:** a College of Medicine and Health, University College Cork, Republic of Ireland; b National Heart Centre, Singapore; c Department of Pharmacology and Therapeutics, University College Cork, Western Gateway Building, Republic of Ireland

**Keywords:** Blood pressure, Nocturnal dip, Hypertension, Seasonal variation

## Abstract

**Background:**

Normal blood pressure (BP) follows a circadian rhythm, with dipping of BP at night. However, knowledge is limited in how the nocturnal dipping in hypertensive patients changes with the seasons. The study aims to examine the pattern of seasonal changes of nocturnal dip in an Irish population and furthermore, to compare it to the pattern observed near the equator where such seasonal variations are minimal, by also studying a Singaporean population.

**Methods:**

Ambulatory Blood Pressure Monitor recordings were obtained from 220 patients, half were from Mercy University Hospital, Cork, Ireland and half from the National Heart Centre, Singapore during the summer period from May to June and the winter period from October to December.

**Results:**

Irish seasonal changes resulted in an increase in nocturnal dipping in the hypertensive patients, especially for diastolic pressure (95% CI, 0.72 to 6.03, 3.37mmHg; p<0.05) and a change in the duration of dipping at night (95% CI, 0.045 to 1.01, 0.53h; p < 0.05). In Singapore, slight differences in dipping in systolic pressure were apparent despite the presence of only minor alterations in temperature (95% CI, 0.38 to 4.83, 2.61mmHg; P<0.05) or duration of daylight.

**Conclusion:**

Seasonal changes not only affected the daily blood pressure but also the night time dipping status in hypertensive patients by mean value of 1.99mmHg and 3.38mmHg for systolic and diastolic pressure dip respectively. This has implications on how hypertensive patients should be treated during different seasons and when they are traveling to countries of different climatic environment.

## 1. Introduction

Various studies have demonstrated the presence of seasonal variations in BP [1–4]. Alterations in vascular resistance and intravascular volume are thought to play a role in this variation. Bankir et al. have proposed that BP rises in winter due to vasoconstriction and falls in summer due to vasodilation because of the ambient temperature which causes changes in sympathetic activity as well as loss of water and salt through sweating [5]. Circadian variations have also been demonstrated, where BP dips at night. Nocturnal dipping in BP is defined as the normal physiological change in the human body, which causes a 10–15% fall in BP at night time as compared to the day.

This night time dipping is thought to be affected by an inability to excrete sodium during the daytime [6]. Night time dipping has been found to be greatly reduced in populations who are regularly exposed to seasonal changes [7].

Brennan et al. accurately mapped out the changes in blood pressure due to acclimatization in mildly hypertensive people [8]. They demonstrated that mildly hypertensive patients have a difference of 27 mmHg in systolic and 24 mmHg in diastolic pressure between summer and winter season. However, few studies have successfully shown how a change in season may affect the night time dipping status in blood pressure. A study performed by Kario et al. [9], showed that in hypertensive patients, extreme dipper status (more than 20% decrease in night time BP) may be related to silent and clinical cerebral ischemia through hypoperfusion during sleep or an exaggeration in the rise of blood pressure during the day, whereas reverse dippers are at a higher risk for intracranial hemorrhage. This shows the importance in knowing the pattern of dipping during different seasons, with an impact of this knowledge on the choice of the medications but also the treatment for other associated diseases, including obstructive sleep apnea [10].

This study aims to elucidate the seasonal pattern of nocturnal dipping in patients with a clinical diagnosis, or suspicion of hypertension. The research also attempted to take into consideration any secondary features such as seasonal affective disorder and physical exertion both of which vary more in Ireland than Singapore.

By exploring the effect of seasonal variations on the duration of the nocturnal dip we will not only provide clinicians with a better appreciation of the factors that influence the successful control of hypertension but also explore any forms of secondary features such as Seasonal Affective Disorder and physical exertion both of which are affected by the daylight shift.

## 2. Methods

Patients who were referred to Mercy University Hospital, Cork, Ireland and National Heart Centre, Singapore were considered for the study. Hypertensive patients that were selected for inclusion in the study were randomly selected by a non-stratified method. Those that were shown to be taking any other forms of non-anti-hypertensive medications that might alter the BP were excluded from the study.

The data being collected have been ethically approved by the Clinical Research Ethical Committee (CREC) of UCC and the Centralised Institutional Review Board (CIRB) of Singapore. The data were collected from May to October 2014.

Data on systolic blood pressure and diastolic blood pressure are recorded by 24h Ambulatory Blood Pressure Monitor (ABPM). Race, age, gender, Body Mass Index (BMI), any present diagnosis, family history, anti-hypertensive or diuretic drugs that affects blood were recorded from the Citrix system and medical reports in Singapore and Ireland. The Ambulatory Blood Pressure Monitor used in Singapore was a Spacelabs Model 90217 while those used in Ireland were Meditech LTD ABPM-04, Medtech LTD ABPM-05 and Spacelabs model 90207. The MUH ABPM data were analyzed using the DABL software system, DABL Health, Dublin, Ireland. The ABPMs were programmed to record BP at thirty minute intervals over the day time and the night time. Systolic and diastolic ABP and heart rate (HR) values were averaged to each hour of the recording, over the daytime (from 08:00–20:00h), over the night time (from 01:00–06:00h), and over the entire 24-h periods [11].

The retrospective data were collected from the period of May to July as the summer season and October to December as the winter season from 2013. 110 patients’ details were collected from each season amounting to 220 patients per country.

Patients were further divided into hypertensive, which includes, and non-hypertensive patients. Hypertensive patients refers to patients who have a systolic pressure greater than 135mmHg and diastolic pressure of 85mmHg on ABPM. The diagnosis is made according to National Institute for Health and Care Excellence (NICE) guidelines [12].

Data are also expressed in terms of means with standard deviations using descriptive statistics or paired sample T test or independent sample T test is used. The different numbers of each dippers in both countries are compared amongst themselves to find out whether there was any change by using the paired sample T test in SPSS Statistics 20 to obtain the p-value.

Data on temperature, humidity and other meteorological data will be obtained from the Irish Meteorological Service (MET) and from the National Environment Agency (NEA) of the Singapore government.

Data on the electricity consumption by household in Singapore was taken from the Energy Market Authority (EMA) of Singapore.

## 3. Results

### 3.1. Characteristics of patients

A total of 220 patients were studied in Ireland and in Singapore. There were a total of 62 males and 43 females, 82 males and 28 females in Singapore during the summer and winter seasons respectively. In Ireland, there were 64 males and 46 females, 58 males and 52 females during summer and winter seasons respectively. The mean age, in years, in Singapore was 52.29 in summer and 46.37 in winter, In Ireland the mean age in summer was 57.64 and in winter was 55.63. Tables 1 and 2 provide a summary of the characteristics of the population in each country. The two populations involved in the study have relatively similar BMI. In Singapore, the BMI for summer and winter seasons are 28.9 and 26.9 while that in Ireland is 28.11 and 30.25.

### 3.2. Seasonal difference between Ireland and Singapore

**Fig. 1:**
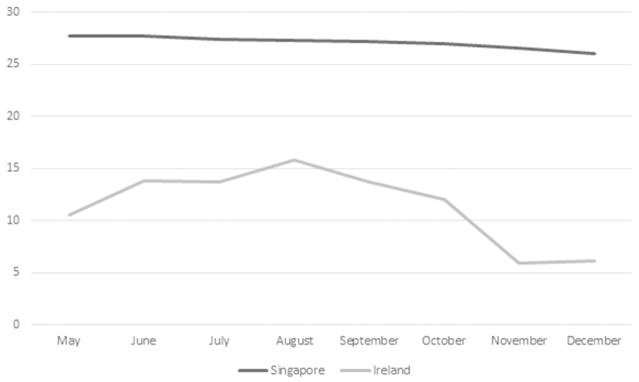
Seasonal variation in temperature in Ireland and Singapore (°C).

**Fig. 2:**
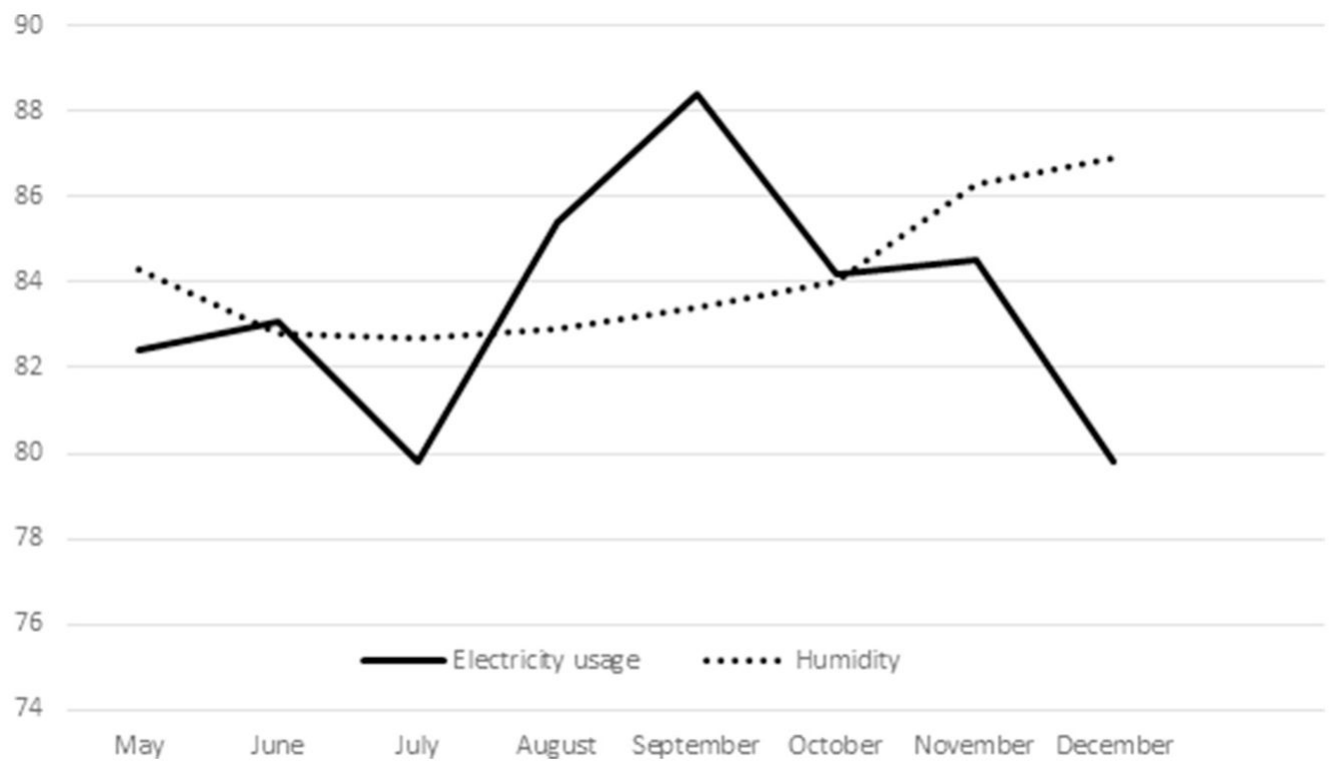
Changes in household usage of electricity (kWh) and humidity (%) in Singapore.

**Fig. 3:**
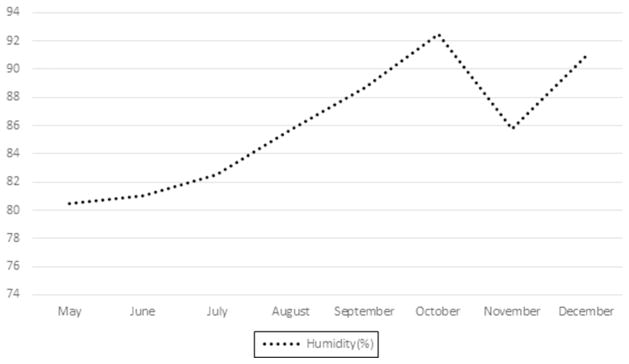
Changes in humidity (%) in Ireland.

Unlike Ireland, Singapore experienced a relative stability of temperature from May to December (Fig. 1). This is because Singapore lies closer to the equator and does not experience any forms of seasonal changes. There is also a relatively stable humidity over these months (Fig. 2). In contrast, Ireland experienced a significant rise in humidity over the periods from May to December (Fig. 3). Singapore also does not experience any form of daylight shift as the number of daylight hours is generally 12h over the whole year, whereas Ireland experiences changes in daylight hours during different seasons. Changes in temperature, humidity and daylight hours all differed between Ireland and Singapore.

### 3.3. Interactions between seasonal changes and blood pressure dipping

During the different seasons, there were changes in mean systolic and diastolic pressure dipping for both Ireland and Singapore as summarized in Table 3. The seasonal difference between Ireland and Singapore exhibited a significant change in diastolic dip of blood pressure (95% CI, 0.72 to 0.72, 6.03mmHg; p=0.013) as well as the duration of night time dipping in Ireland (95% CI, 0.045 to 1.01, 0.53 Hours; p = 0.032) but not in Singapore. Surprisingly, systolic night time dipping in Singapore exhibited significant changes (95% CI, 0.38 to 4.83, p=0.022) whereas a non-significant trend was evident in Ireland (95% CI, 0.42 to 4.40, p=0.105). This could be linked to the greater usage of air-conditioning in Singapore and heating system in Ireland during the changes in temperature. This is associated with changes in electricity usage in Singapore in Fig. 2. However, the relevant data of Ireland is not available. The seasonal changes did not affect the overall number of dippers in each group as changes were noticed in both Ireland and the control group, Singapore.

However, when the average sleeping blood pressure between the two countries in the two seasons were compared (Figs. 1 and 2), it was evident that Singapore did not experience as extensive a change in systolic BP (123.80 to 123.52mmHg), whereas diastolic BP increased instead, from 71.95 to 73.36mmHg). This is in contrast with the Irish average sleeping systolic BP which decreased from 119.14 to 117.21mmHg and diastolic which decreased from 65.82 to 62.55mmHg. This seems to show that despite mean systolic dipping changes in Singapore, the night time sleeping systolic and diastolic blood pressure hover around a constant value whereas that for Ireland changes due to the different seasons. The overall pulse pressure for Singapore decreased from 51.85mmHg to 50.16mmHg between summer and winter while that for Ireland increased from 53.32mmHg to 54.66mmHg due to a greater decrease in diastolic pressure in Ireland.

## 4. Discussion

The results of this study show that there was a seasonal link with the nocturnal dip in hypertensive patients. As seen in Table 4 there was a change in the diastolic dip in Ireland but not in Singapore. This is consistent with the findings of previous researchers who reported a link between seasonal changes in blood pressure due to increased vasodilatation during hot weather. It tends to affect the diastolic pressure more in hypertensive patients. It is estimated by Woodhouse et al. that a 1 °C decrease in living room temperature will increase systolic and diastolic blood pressure by 1.3mmHg and 0.6mmHg respectively [13].

Fig. 1 displays the seasonal change in temperature, which shows that the average change in temperature between summer and winter in Singapore and Ireland are 1.7°C and 7.8°C. This would mean that the actual change of temperature in Ireland is 4.5 times that of Singapore. The change in mean dipping of diastolic blood pressure is also more significant in Ireland (3.4%) as compared to Singapore (2.1%). There was a trend towards a seasonal change in Singapore. This might attribute to the fact that, despite small, there was still a slight decrease in temperature in Singapore over the months from May to December, but may also reflect changes in humidity and possibly in the use of air conditioning.

However, the systolic dip at night in Singapore was an unexpected change (p < 0.05). In fact, the mean systolic dip changed a total of 2.6% as compared to 1.99% of Ireland. This could be due to many other variables which might have a greater effect on the systolic pressure. In the Irish population, there is an overall decrease in pulse pressure from 56.23 to 56.15mmHg while that in Singapore increased from 53.12 to 53.9mmHg.

In addition, the duration of night time dipping between the two seasons in Ireland changed from 2.99 to 3.52h while that in Singapore was only from 2.57 to 2.97h. The result could also be an underestimation of the true value of the dipping as the average age in the Irish population is slightly higher. The average age for the Irish population is 58 and 56 between summer and winter while that in Singapore is 52 and 46. Age is one of the key factor which will increase the frequency of arousal from sleep [14]. This might inevitably decrease the total duration of night time dipping in Ireland.

**Fig. 4:**
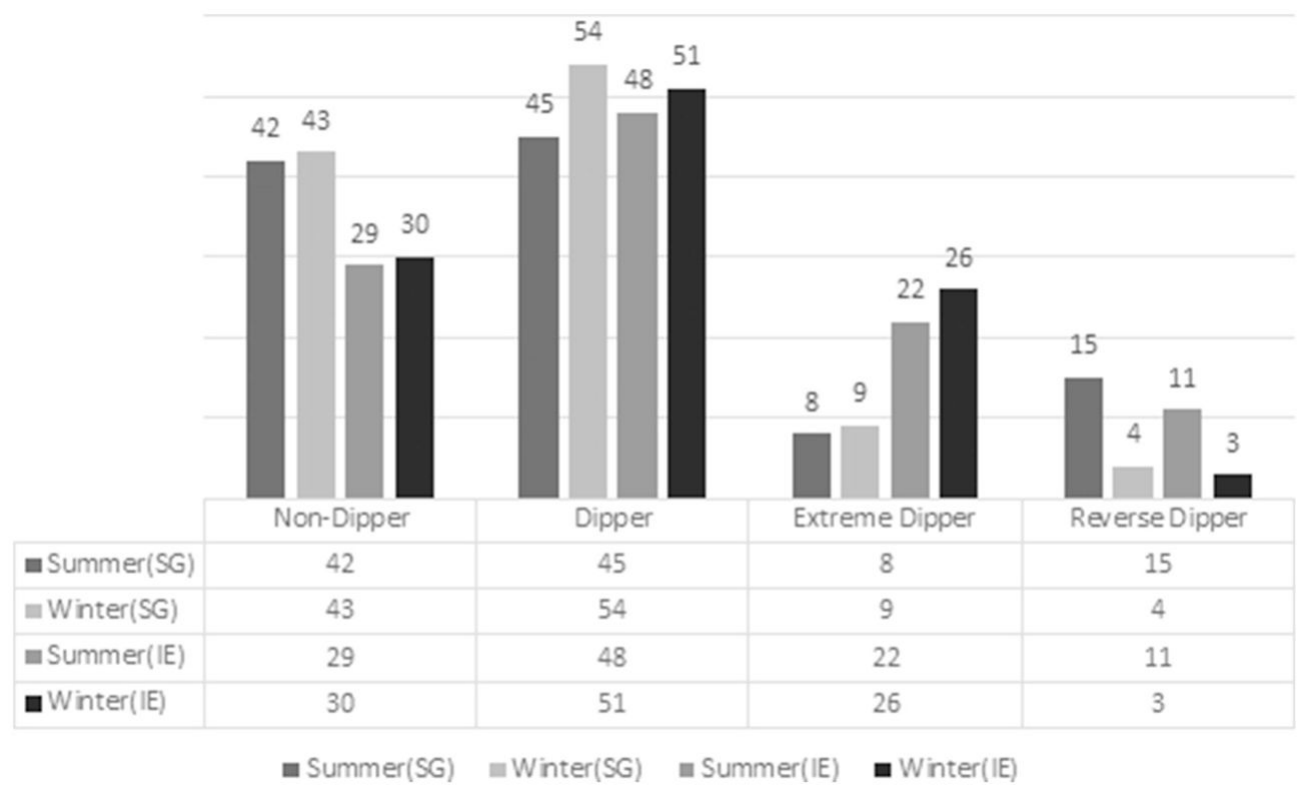
Numbers of different types of dippers during the summer and winter in Ireland and Singapore

Interestingly, even though there are no changes in seasons in Singapore, both Ireland and Singapore seem to show an overall drop in the number of reverse dippers and increase in the other group of dippers (Fig. 4). This would mean the changes in type of dippers between the two seasons in Ireland might not be necessarily caused by the seasonal change.

This study is unique in the inclusion of two countries, Ireland and Singapore, one of which has no seasonal changes. It allows the use of a single country to act as a form of control. Many present studies in similar field only use a single country as a form of comparison. This study thus, allowed us to further test the hypothesis.

### 4.1. Limitations of the study

Some limitation of the study involves the fact that as data are being collected from different countries, the Ambulatory Blood Pressure Monitor (ABPM) being used is different which might give rise to systematic errors. However, both centers used systems of ABPM monitoring that are well validated and should minimize observer bias in the collection of individual blood pressure values.

Both countries are very different in terms of culture, demographics as well as dieting style and that the Asian population tends to be lower in BMI as compared to the Caucasian population. In Singapore, the food consumed is generally healthier and tend to be less salty which might result in a lowering of blood pressure as compared to that of the Irish population. Unlike Ireland, Singapore does not have winter season, thus the amount of food consumption are also generally more constant as people tend to consume greater amount and saltier food during the winter as compared to summer. This would inevitably affect the overall blood pressure as well.

However, despite the differences in diet, race and culture, the difference in BMI between both countries was modest. This will allow the exclusion of these difference affecting any changes in blood pressure between the two populations. This is further enhanced by the similarity in the sexes group being used with males being the predominant one due to the fact males are more susceptible to hypertension and cardiovascular diseases.

The population of hypertensive subjects studied in the summer and winter cohorts in each country differed. This is a reflection of the collection of this data from a real world population. Although it would be preferable to study the same population in both phases this was not practical in view of the clinical demand for ABPM and the need to apply it in an environment of routine care. As the comparison of an entire cohort in the two countries seems to show how the season will affect the dipping status in people, this would perhaps, be amplified if the same cohort were used.

Another limitation will stem from the fact that Singaporeans in general are mostly indoors in air-conditioned areas. During a hotter weather the air-conditioners will be used to reduce temperature. Irish on the other hand, will use heaters during the winter season and reduce the change in temperature due to any seasonal changes. In our population of non-night shift works the nocturnal BP recordings will almost exclusively be recorded in the patients’ homes. As a result, the changes in blood pressure might not accurately reflect the actual changes in the temperature changes due to different seasons as the air-conditioning and heating might lower the difference in changes of temperature due to the changes of weather. This might cause an underestimation of the actual change in blood pressure.

## 5. Conclusion

In summary, it is difficult to obtain a single variable from two separate countries with different cultural influences. However, this research shows a clinical significance in the seasonal changes in nocturnal dip and should be taken into consideration during the treatment of diastolic pressure of hypertensive patients. In the usual practice now, not much of an attention is given to the fact that how the seasonal changes will affect the usual blood pressure changes. By taking the changes into consideration, clinicians will be able to control the changes in blood pressure more effectively especially even when the seasons change through the control of dosages of medications prescribed. The change in blood pressure should also be taken into considerations when the patient travels into countries with different climatic environment.

## Figures and Tables

**Table 1:** Characteristics of subjects investigated in Singapore (n = 110, in each season).

Singapore	Range	Mean	Std. deviation
Average age in summer (yrs)	17–90	52.29	20.57
Average age in winter (yrs)	16–89	46.37	19.18
Body mass index in summer (kg/m^2^)	16.8–47.8	28.9	24.3
Body mass index in winter (kg/m^2^)	15.4–57.0	26.9	5.73
Average awake systolic BP in summer (mmHg)	104.00–167.00	135.36	14.03
Average awake systolic BP in winter (mmHg)	109.00–180.00	138.81	12.91
Average sleep systolic BP in summer (mmHg)	85.00–168.00	123.80	16.40
Average sleep systolic BP in winter (mmHg)	97.00–180.00	123.52	13.68
Average awake diastolic BP in summer (mmHg)	46.00–117.00	80.40	11.31
Average awake diastolic BP in Winter (mm Hg)	60.00–121.00	83.59	11.47
Average sleep diastolic BP in summer (mm Hg)	50.00–101.00	71.95	10.13
Average Sleep diastolic BP in winter (mmHg)	49.00–120.00	73.36	10.56
Duration of night time dip in summer (hours)	0–7.50	2.57	1.92
Duration of night time dip in winter (hours)	0–7.50	2.99	1.77

**Table 2:** Characteristics of subjects investigated in Ireland (n = 110, in each season).

Ireland	Range	Mean	Std. deviation
Average age in summer (yrs)	28–83	57.64	11.84
Average age in winter (yrs)	18–82	55.63	14.54
Body mass index in summer (kg/m^2^)	15.0–49.6	28.11	4.97
Body mass index in winter (kg/m^2^)	18.5–42.3	30.25	5.43
Average awake systolic BP in summer (mmHg)	105.00–183.00	135.71	14.81
Average awake systolic BP in winter (mmHg)	97.00–170.00	136.54	13.07
Average sleep systolic BP in summer (mmHg)	87.00–159.00	119.14	14.93
Average sleep systolic BP in winter (mmHg)	78.00–151.00	117.21	13.29
Average awake diastolic BP in summer (mmHg)	58.00–106.00	78.78	9.53
Average awake diastolic BP in Winter (mmHg)	56.00–102.00	79.17	9.97
Average sleep diastolic BP in summer (mmHg)	47.00–100.00	65.82	9.73
Average Sleep diastolic BP in winter (mmHg)	45.00–87.00	62.55	11.96
Duration of night time dip in summer (hours)	.00–7.50	2.99	1.92
Duration of night time dip in winter (hours)	.00–8.00	3.52	1.74

**Table 3:** Mean systolic and diastolic dip between Singapore and Ireland during summer and winter.

Singapore				Ireland			
Mean systolic dip (%)		Mean diastolic dip (%)		Mean systolic dip (%)		Mean diastolic dip (%)	
Summer	Winter	Summer	Winter	Summer	Winter	Summer	Winter
8.34	10.94	9.90	12.01	11.93	13.92	16.06	19.44

**Table 4:** Comparison of type of dipper, systolic and diastolic dip, duration of night time dip between Singapore and Ireland.

	Mean	Levene test for equality of variance	Std. error	95% confidence interval of the difference		Significance
				Lower	Upper	
Type of dipper during summer and winter in Singapore	.200	0.076	0.12	0.036	.044	0.096
Type of dipper during summer and winter in Ireland	.118	0.054	0.12	0.11	0.35	0.309
Systolic dip at night during summer and winter in Singapore	2.61	0.005	1.13	0.38	4.83	0.022
Diastolic dip at night during summer and winter in Singapore	2.11	0.04	1.20	0.25	4.47	0.08
Systolic dip at night during summer and winter in Ireland	1.99	0.259	1.22	0.42	4.40	0.105
Diastolic dip at night during summer and winter in Ireland	3.37	0.814	1.35	0.719	6.027	0.013
Duration of night time dip between summer and winter in Singapore	0.42	0.280	0.25	0.073	0.91	0.095
Duration of night time dip between summer and winter in Ireland	0.53	0.199	0.25	0.045	1.01	0.032

## References

[1] Hopman R, Remen L (1921). Jaherszeitliche krankheitsbereitschaft, Blutdruckhohe und Jahreszeiten. Z Klin Med.

[2] Rose G (1961). Seasonal variation in blood pressure in man. Nature.

[3] Heller RF, Rose G, Tunstall-Pedoe HD, Christie GS (1978). Blood pressure measurement in the United Kingdom heart disease prevention project. J Epidemiol Community Health.

[4] James GD, Yee LS, Pickering TG (1990). Winter–summer differences in the effects of emotion, posture and place of measurement on blood pressure. Soc Sci Med.

[5] Bankir L, Bochud M, Maillard M, Bovet P, Gabriel A, Burnier M (2008). Nighttime blood pressure and nocturnal dipping are associated with daytime urinary sodium excretion in African subjects. Hypertension.

[6] Fukuda M, Goto N, Kimura G (2006). Hypothesis on renal mechanism of non-dipper pattern of circadian blood pressure rhythm. Med Hypotheses.

[7] Horvath SM, Howell CD, Dill DB, Adolph EF, Wilber CG (1964). Organ systems in adaptation: the cardiovascular system. Adaptation to the Environment.

[8] Brennan PJ, Greenberg G, Miall WE, Thompson SG (1982). Seasonal variation in arterial blood pressure. Br Med J.

[9] Kario K, Pickering TG, Matsuo T, Hoshide S, Schwartz JE (2001). Stroke prognosis and abnormal nocturnal blood pressure falls in older hypertensives. Hypertension.

[10] Wolf J, Hering D, Narkiewicz K (2010). Non-dipping pattern of hypertension and obstructive sleep apnea syndrome. Hypertens Res Off J Jpn Soc Hypertens.

[11] O’Brien E, Asmar R, Beilin L (2003). European Society of Hypertension working group on blood pressure monitoring. European Society of Hypertension recommendations for conventional, ambulatory and home blood pressure measurement. J Hypertens.

[12] National Institute for Health and Core Excellence (2011). Hypertension: clinical management of primary hypertension in adults.

[13] Woodhouse PR, Khaw KT, Plummer M (1993). Seasonal variation of blood pressure and its relationship to ambient temperature in an elderly population. J Hypertens.

[14] Mathur R, Douglas NJ (1995). Frequency of EEG arousals from nocturnal sleep in normal subjects. Sleep.

